# *MtNIP5;1*, a novel *Medicago truncatula* boron diffusion facilitator induced under deficiency

**DOI:** 10.1186/s12870-020-02750-4

**Published:** 2020-12-09

**Authors:** Sara Granado-Rodríguez, Luis Bolaños, Maria Reguera

**Affiliations:** Departamento de Biología, Universidad Autónoma de Madrid, c/Darwin 2, Campus de Cantoblanco, 28049 Madrid, Spain

**Keywords:** Boron deficiency, *Medicago truncatula*, Aquaporins, MtNIP5;1, Boron transport, Legumes

## Abstract

**Background:**

Legumes comprise important crops that offer major agronomic benefits, including the capacity of establishing symbiosis with rhizobia, fixing atmospheric N_2_. It has been proven that legumes are particularly susceptible to boron (B) stress, which leads to important yield penalties. Boron (B) deficiency or toxicity in plants causes the inhibition of growth and an altered development. Under such conditions, the participation of two distinct protein families (the major intrinsic protein family MIP and the Boron transporter family BOR) is required to minimize detrimental effects caused by B stress. However, in legumes, little is known about the transport mechanisms responsible for B uptake and distribution, especially under deficiency.

**Results:**

A *Medicago truncatula* protein, MtNIP5;1 (Medtr1g097840) (homologous to the *Arabidopsis thaliana* AtNIP5;1) was identified as a novel legume B transporter involved in B uptake under deficiency. Further analyses revealed that this *M. truncatula* aquaporin expression was boron-regulated in roots, being induced under deficiency and repressed under toxicity. It localizes at the plasma membrane of root epidermal cells and in nodules, where B plays pivotal roles in symbiosis. Furthermore, the partial complementation of the *nip5;1–1 A. thaliana* mutant phenotype under B deficiency supports a functional role of MtNIP5;1 as a B transporter in this legume model plant.

**Conclusions:**

The results here presented support a functional role of MtNIP5;1 in B uptake under deficiency and provides new insights into B transport mechanisms in legume species.

## Background

Boron (B) is an essential micronutrient for plants as it plays a structural role in plant cell walls, crosslinking two pectin polysaccharides rhamnogalacturonan-II [1, 2]. When B concentrations are below optimum, B-deficiency symptoms appear and include the inhibition of root elongation and a reduced leaf expansion and fertility, which results in substantial agronomical losses around the world [3–5].

In soils, B is mainly found as uncharged boric acid [B (OH)_3_] (Ka = 5.80X10–10 at 25 °C (pKa = 9.24)) [6]. At optimal concentrations, B enters the plant through passive diffusion. However, under deficiency, B is transported into and within the plant by the coordinated action of proteins that belong to two protein families: the major intrinsic protein family MIP and the Boron transporter family BOR [7–10]. MIPs are a superfamily of aquaporins made up of several subfamilies, including the nodulin-26-like intrinsic proteins (NIPs) [11], which are permeable channels to small solutes including [B (OH)_3_] [12–16].

In plants, the best characterized NIP protein described as a B facilitator transporter is the *Arabidopsis thaliana* (L.) Heynh. NIP5;1, (AtNIP5;1), a cell membrane channel permeable to water, urea and [B (OH)_3_] [17]. AtNIP5;1 is found in epidermal cells of roots, and its transcripts are up-regulated under low B conditions, increasing B permeability and maximizing B uptake from B-deficient soils [15].

On the other hand, BOR proteins constitute a family of boric acid and borate exporters, with seven members identified in *A. thaliana* (AtBOR1–7) [18, 19]. AtBOR1 accumulates in the root stele under low-B conditions, and is responsible for xylem loading of [B (OH)_3_], allowing B distribution to the rest of the plant [7, 18]. It has been proven that the coordinated action of AtNIPs and AtBORs is essential to ensure a proper B distribution within the plant, especially under low-B conditions, what is required for a proper *plant* growth and development [20].

Various crops of agronomical interest belong to the legume family (*Leguminosae* or *Fabaceae*), such as alfalfa, pea, or soybean [17, 21]. These plant species have the ability to interact symbiotically with N_2_-fixing Rhizobia, triggering the formation of root nodules, where biological nitrogen fixation takes place [22–24]. In this sense, developing more efficient nitrogen-fixing associations is of high importance given the pollution problems associated with the excessive use of chemical fertilizers [25, 26]. This requires a better understanding of the factors that influence the success of the symbiotic process, including nutrient transport [27–29]. In line with this, it has been shown that every step in the establishment of Legume-rhizobia symbioses is severely affected by B deficiency [30–35]. However, B transport has been barely studied in legumes and the MIP protein superfamily seems to harbor putative B transporter candidates. In the model legume *Medicago truncatula* Gaertn, this superfamily comprises 46 MIPs, 18 of them belonging to the NIP family [36]. However, only one B transporter (a NIP protein) has been characterized so far [37], and none have been associated with B transport under B deficient conditions.

In the present study we use a candidate gene approach combined with a phenotypic analysis and a heterologous complementation assay to characterize MtNIP5;1 as a novel B transporter of *M. truncatula*, homologous to AtNIP5;1, that functions under low boron conditions. The results presented here will allow gaining further knowledge regarding B nutrition in leguminous plants.

## Results

### Boron stress causes developmental and growth defects in *M. truncatula*

In order to study *M. truncatula* response to B availability, a growth assay was carried out applying a B gradient. The plant phenotype and nitrogenase activity (for those plants growing under symbiotic conditions) were analyzed.

**Fig. 1:**
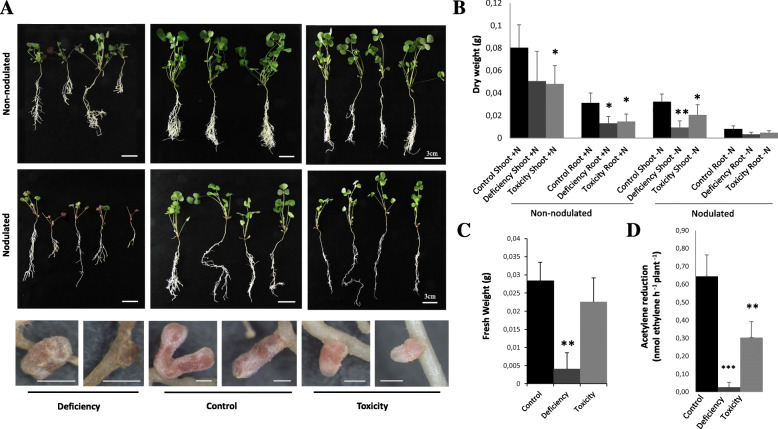
Boron stress has an inhibitory effect on *M. truncatula* growth and nitrogenase activity. **a** Pictures of 4-week-old *M. truncatula* plants growing under a B gradient are shown. Upper panels (upper row of images) show plants fertilized with N (non-nodulated plants) growing under B deficiency (no B was supplemented into the media), control (media at a final B concentration of 0.1 mM B [OH_3_]) or B toxic conditions (1 mM B [OH_3_]). Middle row of images shows plants growing under symbiotic conditions (*M. truncatula* plants inoculated with *S. meliloti* 2011). Lower row of images shows nodules from plants grown under different B conditions, as indicated in the bottom labels. Bar = 3 cm (plant pictures) or 1 mm (nodule pictures). **b** Plant biomass as dry weight of roots and shoots of non-nodulated or nodulated plants under a B gradient. **c** Nodule fresh weight under a B gradient. Data are the Mean ± SD of two independent experiments with, at least, four biological replicates (*n* = 4). **d** Nitrogenase activity was determined in 4-week-old plants growing under a B gradient. Plants growing under control, B deficiency, or B toxic conditions were used to evaluate nitrogenase activity changes under different B conditions. Nitrogenase activity was analyzed by the acetylene reduction method (nmol ethylene generated per hour per plant). Data were analyzed using One-way analysis of variance (ANOVA) followed by t-student *post-hoc* test. Asterisks indicate significant differences when comparing B stress samples with B control treatment (*t*-Student, (“*” = *p < 0.05*, “**” = *p < 0.01,*, “***” = *p < 0.001*)

Plants growing under B deficiency showed chlorosis and the inhibition of leaf expansion and root growth. Generally, these symptoms were more evident in nodulated plants than in those fertilized with N (Fig. 1). Fewer and smaller nodules appeared in these plants compared to those growing under control conditions (Fig. 1). On the other hand, plants growing under B toxicity showed a comparable growth inhibition under symbiotic and non-symbiotic conditions. Generally, nodules of plants growing under B toxicity presented similar sizes to control nodules although a significant number of nodules appeared with smaller size. Biomass data supported most of these observations (Fig. 1a-c). Biomass reduction (of approximately 60%) was observed in roots of both N-fertilized and nodulated plants growing under B deficiency (Fig. 1b). A reduced nodule biomass was also found under B deficiency (Fig. 1c). Under B toxicity, plants showed a decrease in shoot and root biomass under symbiotic and non-symbiotic conditions (Fig. 1b). Nonetheless, nodules did not show an altered biomass under high B levels (Fig. 1c).

Nitrogenase activity was determined and expressed per plant (Fig. 1d) or relative to nodule number or nodule weight (Additional file 2), giving all similar results. Nodules of both B-deficiency and B-toxicity growing plants showed a significant reduction of nitrogenase activity. These results were consistent with the nodule defects observed under B deficiency (Fig. 1a). Interestingly, even though no significant differences were observed under B toxicity in nodule biomass, N fixation was severely affected under these conditions.

### Identification of AtNIP5;1 homologs in *M. truncatula*

**Fig. 2:**
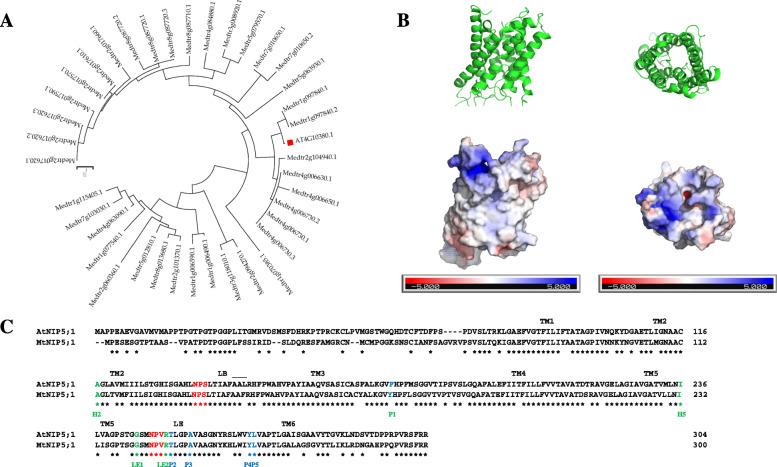
*M. truncatula* MtNIP5;1 is the closest homologous protein to the *A. thaliana* AtNIP5;1, an aquaporin involved in B transport under deficiency. **a** Unrooted phylogenetic tree of *M. truncatula* homologs to AtNIP5;1 (which is indicated by a red square) was obtained using Mega7 software. The tree is drawn so that branch length separating any two sequences is proportional to evolutionary distances. **b** MtNIP5;1 protein predicted model. Upper panel within panel B indicates the tertiary structure model of MtNIP5;1 showing yellow areas that represent the NPS/NPV motifs. Distribution of positive (blue) and negative (red) charges are shown below. The location of positively charged residues in the external part of the pore leaving the negative charges in the inside is shown. **c** Alignment of AtNIP5;1 and MtNIP5;1 protein sequences. Shaded in grey appear the six transmembranous helices and marked in yellow, the regions that form the seventh helix. The NPA motifs are shown in red, residues from the Ar/R selectivity filter appear in green and the five amino acids that shape the Froger’s positions are shown in blue

The *A. thaliana NIP5;1*, encoding an aquaporin that belongs to the MIP superfamily, was the first aquaporin discovered involved in boron transport in plants [15]. Sequence comparison of the encoded protein AtNIP5;1 (AT2G47160), showed that Medtr1g097840 (named MtNIP5;1) is the closest homolog to the *A. thaliana* B transporter in *M. truncatula*, showing 73.7% sequence identity (Fig. 2a).

Aiming to further characterize the MtNIP5;1 protein, a 3D protein structure prediction was performed as described in the Methods section. The predicted tertiary structure indicated that the encoded protein would be transmembranous, presenting six membrane-spanning helices, connected through loops, and two short helices connected via conserved NPS/NPV motifs, together forming the seventh transmembrane helix (Fig. 2b). The distribution of these seven helices would constitute a pore, a characteristic feature of aquaporin proteins [38]. Besides, the protein charge layout indicated the location of positively charged residues in the external part of the pore, leaving the negative charges in the inside (Fig. 2b). On the other hand, a comparison between the amino acid sequences of MtNIP5;1 and its closest homolog in *A. thaliana*, AtNIP5;1, revealed that they share the dual NPAs motifs (being NPS/NPV) and the Ar/R selectivity filter (A-I-G-R) sequences as well as four of the five amino acids (P2-P5) that belong to the P1-P5 Froger’s positions (being Y/F-T-A-Y-L, for AtNIP5;1 and MtNIP5;1, respectively) (Fig. 2c). This supports a shared role between these two proteins transporting boric acid across the plasma membrane [36, 39, 40]..

### MtNIP5;1 expression is induced under B deficiency

**Fig. 3:**
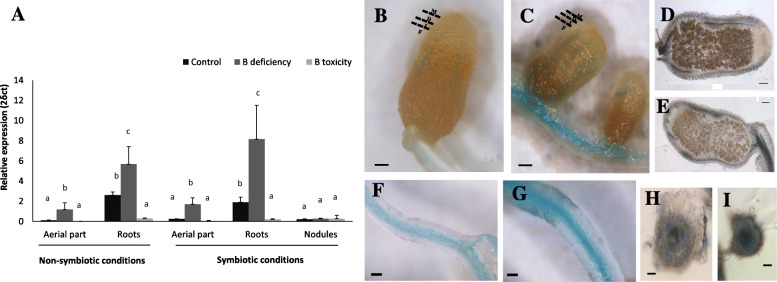
*MtNIP5;1* expression is induced under B deficiency. **a** mRNA accumulation was quantified by qPCR in roots, shoots, and nodules of plants fertilized with N or growing under symbiotic conditions. Plants grew under a B gradient consisting of the following treatments: control conditions (0.1 mM B [OH_3_]), deficiency (0 mM B [OH_3_]), or B toxicity (1 mM B [OH_3_]). Data are the Mean ± SD of two independent experiments with, at least, four pooled plants and four biological replicates (*n =* 4). Different letters indicate significant differences (One-way ANOVA followed by Tukey test, *p < 0.05*). *MtNIP5;1* expression is presented as expression relative values to the internal gene *Ubiquitin carboxyl-terminal hydrolase 1*. **b-i** Tissue expression localization of *pMtNIP5;1::GUS* appears at the vasculature and is induced under B deficiency. Blue GUS staining analysis in *pMtNIP5;1::GUS* appears in the vasculature of **(b-e)**: nodules and **(f-i)**: roots. **b**, **d**, **f,** and **h** show B control treatment (0.1 mM B [OH_3_]), (**c**, **e**, **g,** and **i)**: B deficiency treatment. M: meristematic zone, D: differentiation zone, I: interzone F: N_2_ fixation zone. Bar = 0.5 cm

A qPCR assay was performed in three different tissues (roots, nodules, and aerial parts of nodulated and non-nodulated plants) in order to evaluate possible changes in the expression patterns of *MtNIP5;1* under a B gradient. *MtNIP5;1* expression was induced under B deficiency in both shoots and roots, although this induction was more pronounced in the latter. Besides, the expression was repressed in roots under B toxicity compared to control conditions. Nodules presented lower transcript levels compared to shoots and roots, and the induction did not change significantly among the three B conditions tested (Fig. 3a). In silico analysis based on the Symbimics database [41] or the *Medicago truncatula* Gene Expression Atlas ( [42], ID Mtr.34598.1.S1_at) supports these results, showing much higher expression in roots compared to other plant organs (including nodules or the shoot/aerial part) (Additional file 3).

Going further, the tissue expression of this gene was evaluated by fusing the β-glucuronidase gene (*gus*) to the *MtNIP5;1* promoter. *M. truncatula* seedlings were transformed with a construct containing the 2 kb region upstream of *MtNIP5;1* fused to *gus*. The blue GUS staining was detected in roots and nodules of control and B-deficient plants 4 weeks postinoculation (Fig. 3b). As indicated by qPCR, *MtNIP5;1* was highly expressed in roots, especially under B deficiency. More specifically, the activity was located at the root vasculature. Nodules also presented GUS activity located in the vasculature zone, with a more intense signal under B deficiency (Fig. 3b).

### MtNIP5;1 localizes at the plasma membrane in the epidermis of roots and in the epidermal and cortical cells of nodules

**Fig. 4:**
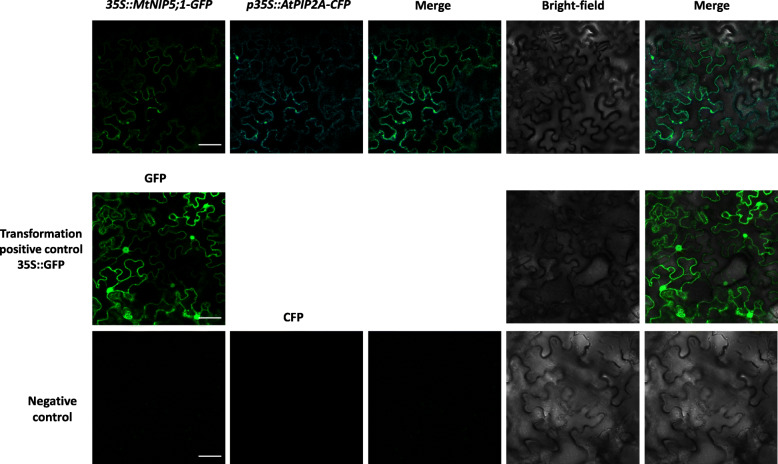
Subcellular localization of MtNIP5;1 in N. benthamiana leaves evidences its localization at the plasma membrane. Left panel shows the GFP signal of MtNIP5;1-GFP, left middle panel shows CFP signal of AtPIP2A-CFP, middle panel shows GFP and CFP merged images, right middle panel shows the bright field image, and right panel the merge between bright-field, GFP signal, and CFP signal. *p35S::MtNIP5;1-GFP* and *p35S::AtPIP2A-CFP* were co-transformed into *N. benthamiana* leaves. The middle row shows *N. benthamiana* cells transformed with *p35S::GFP* as a transformation positive control. The bottom row shows the negative control images obtained from non-transformed leaves*.* Bar = 50 μm

Transient transformation of *Nicotiana benthamiana* Domin leaves was performed aiming to analyze the subcellular location of MtNIP5;1. *Agrobacterium tumefaciens* containing either *p35S::MtNIP5;1-green fluorescent protein (GFP)* or *p35S::AtPIP2A-cyan fluorescent protein (CFP)* constructs were co-infiltrated as described in the Methods section. An overlapping signal was observed between MtNIP5;1 fused to the GFP fluorophore and the plasma membrane marker, indicating that MtNIP5;1 is located at the plasma membrane (Fig. 4). Controls in which no fluorophores were used, showed no signal in the measured channels (Fig. 4).

**Fig. 5:**
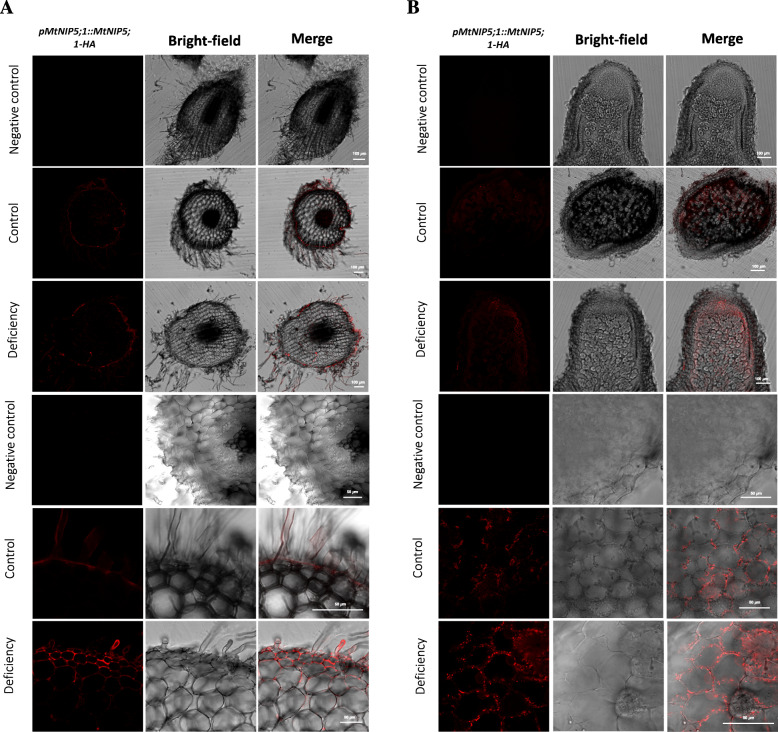
Inmunolocation of MtNIP5;1 in the root epidermis and nodules of ***Medicago truncatula.*** **a** Subcellular localization by immunostaining of MtNIP5;1-HA with the antibody Alexa 594 was performed in root sections of 4-week-old *M. truncatula* expressing *p35S::MtNIP5;1-HA* (red, DsRed channel) under different B conditions (control and deficiency). The three bottom rows show detail of root epidermal and hair root cells showing MtNIP5;1-HA localization. **b** Subcellular localization by immunostaining of MtNIP5;1-HA with the antibody Alexa 594 was performed in nodule sections of 4-week-old *M. truncatula* expressing *p35S::MtNIP5;1-HA* (red, DsRed channel), inoculated with a *Sinorhizobium meliloti* 2011 strain and growing under different B conditions (control, middle row, and deficiency, bottom row). The three upper rows of images are taken at 10Xmagnification, while the three bottom rows show detail of nodule cortex and fixation zone at 40X magnification, showing MtNIP5;1-HA localization. Left column images show HA signal, middle column shows bright-field images and right column shows the merged images of HA signal and bright field. Upper rows in each panel show autofluorescence controls, in which no anti-HA primary antibody was used. Bars = 100 μm (10X images) or 50 μm (40X images)

The chimeric MtNIP5;1-HA protein was used in the immunohistochemical studies. *M. truncatula* plants were transformed with the genomic region of *MtNIP5;1* fused in-frame with three haemagglutinin (HA) epitopes in the C-terminus (MtNIP5;1-HA), using the same promoter region as the one used for GUS activity analysis. MtNIP5;1-HA was detected (DsRed channel) with a mouse anti-HA antibody and an Alexa594-conjugated anti-mouse secondary antibody. Images showed protein localization at the epidermal cells of roots (including root hairs) (Fig. 5a) under both B conditions (although being slightly higher under deficiency). Besides, in nodules, the signal decreased compared to root epidermis and appeared in some infected and non-infected nodule cells, nodule cortex, and surrounding the vasculature (Fig. 5b). Intriguingly, MtNIP5;1-HA signal distribution was not consistent with the tissue-specific expression observed in the GUS staining assays, where an intense signal appeared located exclusively at the vasculature of roots and nodules (Fig. 3). Controls in which no Alexa 594-conjugated antibody was used showed no signal in the measured channels (Fig. 5).

### Partial complementation of nip5;1 *A. thaliana* mutant supports a role of MtNIP5;1 as a B transporter

Aiming to characterize functionally this putative B transporter, a heterologous expression system in *A. thaliana* was used. Thus, the *A. thaliana nip5;1–1* mutant [15] was transformed with the *p35S::MtNIP5;1-GFP* construct, and two independent homozygous single-copy lines were obtained (*p35S:: MtNIP5;1-GFP* 1 and *p35S::MtNIP5;1-GFP* 2). These lines were used to test MtNIP5;1 capability to uptake B and thus complement the mutant phenotype. WT, *nip5;1–1* and *nip5;1–1* mutant lines overexpressing *MtNIP5;1* were grown under two B conditions, control (100 μM B [OH]_3_) and deficiency (0.03 μM B [OH]_3_).

**Fig. 6:**
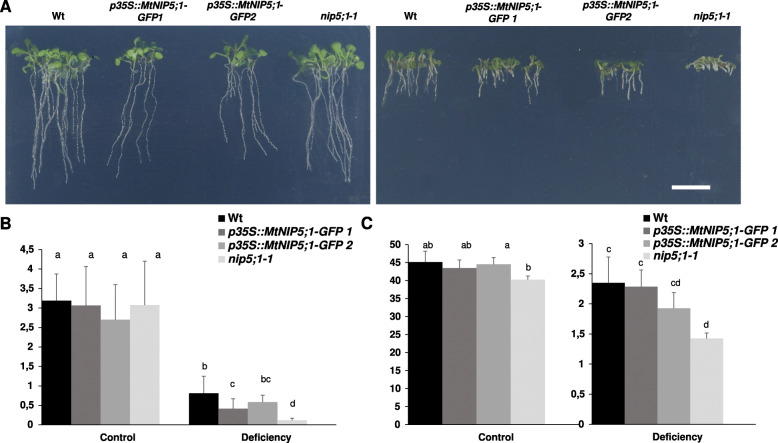
MtNIP5;1 partially complements *nip5;1–1* B deficiency phenotype*.* **a** Images of seedlings (Wild type (Wt), two independent *nip5;1* mutant lines expressing *p35S::MtNIP5;1-GFP* (*p35S::MtNIP5;1-GFP* 1 and *p35S::MtNIP5;1-GFP* 2*),* and *nip5;1–1*) growing under control (left panel) or B deficient conditions (right panel). Bar = 1 cm. **b** Primary root length of Wt (black columns), *p35S::MtNIP5;1-GFP* 1 (dark grey columns), *p35S::MtNIP5;1-GFP* 2 (light grey columns), and *nip5;1–1* (white columns) was measured 10 days postgermination. **c** B concentration was quantified in 10-days old Wt (black), *p35S::MtNIP5;1-GFP* 1 (dark grey)*, p35S::MtNIP5;1-GFP* 2 (light grey), and *nip5;1–1* (white) seedlings grown under B control and B deficiency conditions. The two B treatments used were: control (100 μM B [OH_3_]) and deficiency (0.03 μM B [OH_3_]). Different letters indicate significant differences using a One-way ANOVA analysis followed by Tukey’s test (*p < 0.05*)

A phenotypical analysis was performed in order to evaluate the effects of reduced B availability in root growth over time (Additional file 4). These effects on primary root length were already noticeable at 3 days postgermination, when *nip5;1–1* roots started to show a significantly lower length compared to the Wt and to the two mutant lines overexpressing *MtNIP5;1*. In fact, at the end of the experiment (10 days after germination), both mutant lines overexpressing *MtNIP5;1* tripled the length of the mutant primary root growing under B deficient conditions confirming a partial complementation of the mutation by overexpression of *MtNIP5;1* (Fig. 6 a and b).

Furthermore, in order to evaluate the ability of MtNIP5;1 uptaking B and to determine if the growth rescue observed in the overexpressing lines was correlated with an increase of B, the micronutrient concentration was quantified in 10-days old seedlings growing under control and B deficient conditions. As shown in Fig. 6c, both *A. thaliana MtNIP5;1* expressing lines (*p35S::MtNIP5;1-GFP* 1 and *p35S::MtNIP5;1-GFP* 2) showed a significant increase in B concentration recovering B concentration to Wt levels. On the contrary, calcium (Ca) concentration did not show differences among lines under B deficiency, although it appeared reduced in both overexpressing lines under control conditions (Additional file 5)*.*

## Discussion

Under the context of food security, sustainable agriculture, and climate change, the interest of understating B transport and B stress tolerance in legumes, particularly under symbiotic conditions, has been increasingly higher, aiming to minimize the impact of this nutrient stress on yields [43, 44]. Thus, this work, by using the model legume plant *M. truncatula*, aimed to further understand B transport in legumes, considering that no B transporter involved in B transport under deficient conditions has been characterized till date in this agronomically important plant family.

Together with the energy-dependent high-affinity B transport system constituted by the BOR family, different members of the MIP family possess a role transporting (and therefore, distributing) B in plants [15, 45]. The *A. thaliana* NIP5;1 was the first described aquaporin implicated in the uptake of B from the soil into the roots. AtNIP5;1 acts coordinately with AtBOR1 (identified and characterized a few years earlier) to ensure the uptake and correct distribution of B under deficient conditions [15, 20]. From there, the use of homology-based analysis has allowed the identification of many B transporters in different plant species (including rice, barley, or *M. truncatula*). However, most of these are functionally related to an increased tolerance to high B (i.e. MtNIP3, [22]).

Many different MIP family members, including those that belong to the Tonoplast membrane Intrinsic Proteins (TIP) subfamily (such as AtTIP5;1 [46]) or to the Plasma membrane Intrinsic Proteins (PIP) subfamily (such as the barley Hv-PIP1;3 and Hv-PIP1 [12], the maize ZmPIP1 [47] or the rice OsPIP2;4, OsPIP2;7, OsPIP1;3 and OsPIP2;6 members [48]), have been found to be involved in the transport of B in plants. MtNIP3 was identified as a homologous protein to AtNIP6;1 (sharing 75% of amino acid sequence similarity) in *M. truncatula*, a close relative to the forage legume alfalfa and widely used as a model plant for genetic and genomic studies [49]. MtNIP3 physiological role was related to conferring a high B tolerance, as it showed higher leaf expression levels in B tolerant cultivars, which correlated positively with lower B concentrations in leaves [22].

In the present study, we search for homologs to AtNIP5;1 in *M. truncatula* aiming to identify putative B transporters involved in B transport under B deficiency. The analysis yielded MtNIP5;1 protein as the most promising candidate that may function as AtNIP5;1 based on the amino acid sequence similarity (sharing 73.7% amino acid identity, Fig. 2a). This protein was previously pointed as a putative B transporter homolog to AtNIP5;1 by Bogacki et al. [22] although no further analyses were carried out to prove its functional role, as this work focused on studying the role of another *M. truncatula* NIP protein, MtNIP3 (Medtr4g006730.1), that functions as a B transporter under B toxicity. This previous work together with the results here presented, support distinct roles of NIP proteins in B transport, being involved in toxicity and deficiency responses [14, 15]. Our results showed that B deficiency induces *MtNIP5;1* expression mainly in roots, but also in shoots (Fig. 3a). Furthermore, B toxicity can repress this gene expression specifically in roots, which supports a possible role of this *M. truncatula* NIP protein uptaking B. Besides, this result raises interesting questions regarding *AtNIP5;1* expression regulation by B conditions. Noteworthy, as found in *AtNIP5;1*, the 5′ untranslated region (5′UTR) of *MtNIP5;1* (Additional file 6), may play pivotal roles in controlling the transcript accumulation in response to B conditions [50].

Going further in the analysis, MtNIP5;1 3D structure was predicted. From this analysis, it was inferred that MtNIP5;1 is a transmembrane protein according to its predicted structure, like AtNIP5;1. It presents the characteristic structure conserved among aquaporins (with seven helices with neutral charge distribution) ( [51], Fig. 2b). AtNIP5;1 had been previously described as a transmembrane protein located in the plasma membrane [17]. Besides, the presence of NPA dual motifs (NPS/NPV in this case) in MtNIP5;1 is also a conserved feature among aquaporins, whose function is to facilitate the exit of protons when solutes pass through the channel [52]. Furthermore, aquaporins´ selectivity filter seems to be determined by the amino acids present in the NPA dual motifs, the Ar/R selectivity filter composition, and also the five Froger’s positions (P1-P5) [40, 53]. In the case of the five amino acids located at the Froger’s positions, they may not only be involved in the discrimination between water and glycerol (distinguishing aquaporins from aquaglyceroporins), but also P2-P5 amino acids (and not P1, that is shown not conserved among MIPs for the same substrate) could be relevant discriminating other substrates due to their potential interaction with them [40]. Thus, the high similarity found in these amino acid sequences in MtNIP5;1 and AtNIP5;1 (Fig. 2c) provides further evidence in support of a comparable permeability of non-aqua substrates, such as boric acid [36, 40].

In *A. thaliana*, AtNIP5;1 localizes at the plasma membrane of root epidermal cells functioning as a boric acid channel responsible for B uptake under B limited conditions [15, 17]. In here, protein localization showed similar patterns. MtNIP5;1 appeared localized at the plasma membrane (Fig. 4) and at the root epidermal cells (Fig. 5), what fits well with a role of this facilitator protein ensuring B incorporation. However, an intriguing result was obtained when comparing GUS expression patterns and protein localization analysis as they show differential expression and localization patterns (Figs. 3 and 5). Further analysis should be carried out to investigate these results that resemble those of AtNIP1;1, an AtNIP5;1 homolog protein, what may indicate a cell type-specific posttranscriptional regulation of these aquaporins, including *MtNIP5;1* [54].

Interestingly, expressing *MtNIP5;1* under the control of the cauliflower mosaic virus 35S RNA (CaMV 35S) promoter complemented partially root growth defects shown by *nip5;1–1* under B deficiency (0.03 μM B [OH3]) (Fig. 6), and this partial phenotype rescue was well correlated with a higher B content in the overexpressing lines (Fig. 6c). These results suggest, on one hand, that *MtNIP5;1*-GFP is functional *in planta* and on the other, that *MtNIP5;1* shares functional similarity with AtNIP5;1. Therefore MtNIP5;1 is the first characterized B transporter acting under B deficiency in legumes. In this regard, several reports have shown the symbiosis reliance on B [31, 33–35, 55]. In here, when plants were subjected to deficiency, a characteristic B stress response was observed in nodulated plants, consisting in a lower nodule number, which were smaller in size and brownish and with a reduced nitrogenase activity (Fig. 1). This supports a role of B in nitrogen fixation (Fig. 1d) [30, 34, 35]. In fact, it was initially hypothesized that MtNIP5;1 could play a distinctive role in transporting B to nodules under deficiency. However, and despite observing that MtNIP5;1 localizes to this organ (Fig. 5b), the lack of induced expression under deficiency (Fig. 3a) does not support a unique role of this transporter in B uptake under deficiency in nodules. Nonetheless, it still cannot be ruled out a helper role of this aquaporin in uptaking B under B limiting conditions in nodules.

## Conclusions

Overall, the studies here performed aimed at characterizing putative B transporters of *M. truncatula* belonging to the aquaporin NIP subfamily, functioning under B deficiency. A homology-based analysis pointed to MtNIP5;1 as the most promising candidate to function as AtNIP5;1 in this legume model plant. The induced expression under B deficiency and the repression under B toxic levels, together with this protein localization patterns and the partial complementation of *nip5;1* mutant, support a role of MtNIP5;1 functioning as a B transporter under B deficiency. These results might help better understanding B transport in legumes, what could help developing tolerant cultivars better adapted to B deficient soils. In line with this, it would be interesting to investigate further if legume tolerant varieties present higher expression of this *MtNIP5;1* (or homologous candidates), as has been done with other aquaporins in *M. truncatula* or barley, in which it was linked the tolerance to B toxicity with gene function.

## Methods

### Plant growth conditions

Seeds of *M. truncatula* ecotype R108, obtained from the Noble Research Institute, were scarified with sulfuric acid (H_2_SO_4_), sterilized with bleach before they were germinated in 0.8% agar plates, and placed in the dark at 4 °C for 48 h, following the steps described by Tejada-Jiménez et al. [56]. After stratification, plates were moved to a growth chamber at 22 °C for 24 h with a 16 h:8 h light:dark cycle. Seedlings used for phenotypic or RT-qPCR analyses were transferred to pots using perlite as a substrate and grown in the greenhouse with a long-day photoperiod (16 h:8 h light:dark cycle at 18–25 °C). Greenhouse plants were irrigated every 2 days with Jenner’s solution or water, alternatively [57]. Jenner’s solution was supplemented or not with B (to achieve control or B deficient conditions, respectively) and N (to achieve non-symbiotic or symbiotic conditions).

*N. benthamiana* plants, obtained from Dr. González-Guerrero’s Laboratory (CBGP-UPM/INIA, Spain), were pre-germinated in peat for 10–15 days at 20–22 °C with a 16 h:8 h photoperiod. They were then transplanted to peat:vermiculite 3:1 and grown for 3–4 weeks in a greenhouse at 18–25 °C and long-day photoperiod.

*A. thaliana* ecotype Col-0 and *nip5;1–1* mutant seeds [58], obtained from Dr. Miwa Laboratory (Hokkaido University, Japan), were sterilized first in ethanol 70% and then in bleach 50% with a droplet of Tween-20. After rinsing well the seeds with H_2_O_d_, they were kept overnight in the dark at 4 °C and then germinated in plates with half-strength MS medium [59]. For segregation experiments, seedlings were transplanted to peat:perlite 3:1 and grown in a growth chamber at 23 °C with long-day photoperiod.

### Phenotypic analysis

In order to study B-availability effects on plant growth, inoculated and non-inoculated *M. truncatula* plants were irrigated with different concentrations of B [OH]_3_. Control treated plants were irrigated with 0.1 mM B [OH]_3_, toxicity conditions were achieved by irrigating plants with a final B [OH]_3_ concentration of 1 mM, and no B [OH]_3_ was applied for deficiency conditions. All irrigation media was treated with Amberlite® IRA743 to eliminate B traces.

Plants grown under symbiotic conditions were inoculated with *Sinorhizobium meliloti* FSM-MA strain [60] without receiving any external input of N (−N). Non-inoculated plants were supplemented with 20 mM NH_4_NO_3_ (+N).

Plant tissue was collected 5 weeks after the transplant (4 weeks after inoculation) for biomass measurements, nitrogenase activity, and RT-qPCR assays.

Biomass as fresh weight was determined immediately after plant harvesting. Biomass as dry weight was determined after drying the plant tissue at 60 °C for 72 h.

Nitrogenase activity was measured using the acetylene reduction assay as described by Hardy et al. [61]. Briefly, roots of nodulated plants were placed in 30 ml vials where 3 ml of air were replaced with 3 ml of acetylene. After 30 min, 4 replicates of 0.5 ml each were extracted and their ethylene content was measured using a gas chromatograph Shimadzu GC-2014 (Japan). A dilution of ethylene and acetylene 0.413 mg/l was used as standard. At the end, nodules were counted and weighed.

### Gene candidate identification

To identify *AtNIP5;1* homologous genes that putatively encode B facilitator transporters in *M. truncatula*, the *AtNIP5;1* (*At4g10380*) sequence was obtained from the TAIR database [62] and BLAST in the *M. truncatula* genome using the Phytozome database [63].

### Gene expression analysis by RT-qPCR

Gene expression studies were carried out by real-time RT-PCR (Applied Biosystems®) in order to analyze transcript levels of candidate genes. RNA extraction was carried out using the RNeasy Mini Kit (Qiagen). cDNA was obtained from 500 ng of DNA-free RNA using PrimeScript™ (Takara Bio Inc., Japan). Primers used are indicated in Additional file 1. RNA levels were normalized by using the *ubiquitin carboxy-terminal hydrolase* gene as an internal standard for *M. truncatula* expression patterns [64].

### *M. truncatula* transformation

The *Agrobacterium rhizogenes* ARqua1 strain containing the vector was used to transform *M. truncatula* seedlings 18 h after germination in a growth chamber at 22 °C. Transformation experiments were performed following the protocols described by Boisson-Dernier et al. [65]. Transformed seedlings were later transferred to Farhaeus media plates supplemented with kanamycin (50 μg/ml) as a selection marker [66]. After 3 weeks, plants were transplanted to sterile perlite pots that were placed in the greenhouse at 18–25 °C and long-day photoperiod.

### GUS staining

The *MtNIP5;1* promoter::β-glucuronidase (GUS) construct was generated using the Gateway System (Life Technologies, Carlsbad, USA). The promoter fragment of the candidate gene (2022 kb upstream of the *MtNIP5;1* start codon, P_-1174 UT848_) was amplified using the primers indicated in Additional file 1, cloned in the pDONOR27 vector (Invitrogen), and transferred to the pGWB3 plasmid [67].

Gus activity assay was performed in plants 4 weeks after inoculation following the protocol described by Vernoud et al. [68] with minor modifications. Roots and nodules sections (100 μm) were incubated in a GUS buffer (0.69% PO_4_H_2_Na, 0.5 M EDTA pH 8, 30% sarkosyl, 40 μl triton X-100, and H_2_O) supplemented with X-Gluc (0.1 mg/ml) for 12-16 h at 28 °C in the dark, and then distained with bleach 50% and rinsed five times in H_2_O. Afterwards, sections were observed with a Leica DM IRB microscope.

### Immunohistochemistry and confocal microscopy

*MtNIP5;1* gene and its native promoter (2 kb upstream of the start codon) were amplified with the primers indicated in Additional file 1 and cloned into the plasmid pGWB13 that fuses three C-terminal hemagglutinin (HA) tags in-frame using the Gateway system (Life Technologies, Carlsbad, USA).

Transformed plants were inoculated with *S. meliloti* 2011. Roots and nodules were fixed overnight in a 4% paraformaldehyde, 2.5% sucrose, and PBS buffer at 4 °C. After several washes in PBS, the tissue was embedded in 6% agarose and 100 μm sections were prepared in a Vibratome 1000 plus. Sections were dehydrated using methanol series (30, 50, 70, 100% in PBS) for 5 min and then rehydrated. The immunostaining was started permeabilizing plant cell walls with 4% cellulase in PBS for 1 h at RT and with 0.1% Tween 20 in PBS for 15 min. Sections were blocked with 5% bovine serum albumin (BSA) in PBS before their incubation with anti-HA mouse monoclonal antibody (Sigma) for 2 h at room temperature. After several washes, sections were incubated for 1 h with Alexa594-conjugated anti-mouse rabbit monoclonal antibody (Sigma). Images were acquired using a confocal laser-scanning microscope (Leica SP8) at 561 nm for Alexa 594 imaging using identical settings in each magnification (10X or 40X) in order to make qualitative comparisons among tissues (roots and nodules under control or deficient conditions).

### *N. benthamiana* transient expression assay

The *MtNIP5;1* gene was amplified using the primers indicated in Additional file 1, and then cloned into the pGWB5 plasmid, which fuses CaMV 35S promoter and the GFP tag C-terminally [67]. *A. tumefaciens* C58C1 [69] was transformed with either *p35S::MtNIP5;1-GFP* or the construct *p35S::AtPIP2A-CFP* [70] together with the silencing suppressor p19 of the Tomato bushy stunt virus [71]. *N. benthamiana* 3 weeks-old leaves were then infiltrated following the protocol described by Sparkes et al. [72]. Images were taken 48 h after agroinfiltration with a confocal microscope (Leica SP8).

### Bioinformatic analysis

The aminoacidic sequence of the gene candidate was obtained from the database UniProt [73]. Protein 3D models were predicted using I-TASSER [74] and were edited with the software PyMol.

### *A. thaliana* mutant complementation assay

*A. tumefaciens* strains containing the construct *p35S::MtNIP5;1-GFP* were used to transform the *nip5;1–1 A. thaliana* mutant [58] using the floral dipping transformation method as described by Zhang et al. [75]. Homozygous lines containing the construct of interest were selected by kanamycin selection and PCR analysis, using the primers included in Additional file 1.

Seeds of *A. thaliana* Col-0 (wildtype, Wt), the mutant *nip5;1–1*, and two homozygous independent lines incorporating the construct *p35S::MtNIP5;1-GFP* were grown vertically in plates of ½ MS medium with two B treatments: control (100 μM B [OH]_3_) and deficiency (0.03 μM B [OH]_3_). Primary root length was then measured at 3, 5, 7, and 10 days post-germination, from the root tip to the hypocotyl boundary, using the online available software ImageJ [76].

B concentration was analyzed following Gómez-Soto et al. [77] methodology. Briefly, a pool of seedlings (40–50 seedlings) were collected 10 days after germination and dried out at 65 °C. Dried samples (using three replications per line and treatment) were then submitted to the Elemental Analysis Unit at the Interdepartmental Investigation Service Laboratory at the Universidad Autónoma de Madrid (SIdI-UAM, Madrid, Spain). Plant dry matter underwent acid digestion in a microwave oven and was later analyzed using the ICP-MS NexION 300XX (Perkin Elmer Inc., Hopkinton, MA, USA), as described by Reguera et al. [78].

### Statistical analysis

One-way analysis of variance (ANOVA) followed by a Tukey HSD *post-hoc* test was applied to perform multiple comparisons at a probability level of 5% (*p < 0.05*). The SPSS Statistics 17.0 (SPSS Inc.) package was used for the statistical analyses.

## Supplementary Information

**Additional file 1: Table S1** Primer list.

**Additional file 2: Fig. S1**. Nitrogenase activity was determined in 4-week-old plants growing under a B gradient: control conditions (media at a final B concentration of 0,1 mM B[OH3]), B deficiency (no B supplemented into the media), or B toxic conditions (1 mM B[OH3]). Nitrogenase activity was analyzed by the acetylene reduction method and expressed as nmol of ethylene generated per hour per nodule number (left panel) or per nodule weight (right panel). Data are the Mean ± SD of two independent experiments with, at least, four pooled plants and four biological replicates (*n* = 4). Asterisks indicate significant differences when comparing B stress plants with B control treated plants (t-Student, “*” = *p* < 0.01, “**” = *p* < 0.001).

**Additional file 3: Fig. S2**. Expression of *Medicago truncatula* MtNIP5;1 (Medtr1g097840) in different plant organs. A) Data obtained from the Symbimics database (https://iant.toulouse.inra.fr/symbimics/). B) Data obtained from the Medicago Gene Expression Atlas (https://mtgea.noble.org/v3/).

**Additional file 4: Fig. S3**. Time cource experiment showing primary root growth of *A. thaliana* seedlings in *nip5; 1–1* complementation assays. Primary root growth (mm) of Wild type (Wt) (black line), two independent lines expressing *p35S::MtNIP5;1-GFP* construct (*p35S::MtNIP5;1-GFP 1 and p35S::MtNIP5; 1-GFP 2,* dark and light grey lines, respectively), and *nip5; 1–1* (dashed line) were measured 3,5,7 and 10 days postgermination. Seedlings were grown under two B treatments: **A**) control (100 μM B[OH], and **B)** deficiency (0.03 μM B[OH3]

**Additional file 5: Fig. S5**. Calcium (Ca) concentration was quantified in 10-days old Wild Type (Wt) (black), *p35S::MtNIP5; 1-GFP 1* (dark grey), *p35S::MtNIP5; 1-GFP 2* (light grey), and *nip;1–1* (white) seedlings grown under B control and B deficiency conditions. The two B treatments used were: control (100 μM B[OH3] and deficiency (0.03 μM B[OH3. Different letters indicate significant differences using a One-way ANOVA analysis followed by Turkey’s test (*p < 0.05*)*.*

**Additional file 6: Fig. S6**.A)Sequence alignment of the UTR regions of AtNIP5;1 (AT2G47160) and MtNIP5;1 (Medtr1g097840). B) End of the 5’ untranslated region (5′UTR) of MtNIP5;1 (Medtr1g097840) and the initial sequence of the gene (starting from the ATG starting codon).

## Data Availability

All data generated or analyzed during this study are included in this published article (and its additional files). Nonetheless, the datasets used and/or analysed during the current study are available from the corresponding author on reasonable request.

## Abbreviations

B: Boron
N: Nitrogen
MIP: Major intrinsic protein family
BOR: Borate transporter family
NIP: Nodulin-26-like intrinsic protein family
GFP: Green fluorescent protein
CFP: Cyan fluorescent protein
CaMV: 35S cauliflower mosaic virus 35S RNA
