# Supplementary material for: MtNIP5;1, a novel Medicago truncatula boron diffusion facilitator induced under deficiency
Source: BMC Plant Biol. 2020 Dec 9;20:552. doi: 10.1186/s12870-020-02750-4 (PMC7724820; doi:10.1186/s12870-020-02750-4)
Supplement: Supplementary file 1 — Additional file 1: Table S1 Primer list. [file 12870_2020_2750_MOESM1_ESM.pdf]

Table S1 Primer list		
**Cloning**		
Medtr1g097840 promoter 5´ extreme	pMedtr1g097840 Forward	GGGGACAAGTTTGTACAAAAAAGCAGGCTCAAGAGCAACGATATTCATACGAA
Medtr1g097840 promoter 3´ extreme	pMedtr1g097840 Reverse	CCTGACTCCGATTCTGGCATGGATCCGGGGACCACTTTGTACAAGAAAGCTGGGTATCTTTTTTTTTTTTTTTTTCTTTAAAAA
Medtr1g097840 gene 3´extreme without Stop Codon	Medtr1g097840 without Stop Codon	GGGGACCACTTTGTACAAGAAAGCTGGGTAGCGACGGAAGCTCCTAACTG
Medtr1g097840 gene 3´extreme With Stop Codon	Medtr1g097840 With Stop Codon	GGGGACCACTTTGTACAAGAAAGCTGGGTACTAGCGACGGAAGCTCCTAAC
**qPCR**		
Medtr1g097840 Forward	Medtr1g097840 Forward	AGTTGCATCGCTAATTTCTCTGCTG
Medtr1g097840 Reverse	Medtr1g097840 Reverse	TGGTCCTGCTGTTGCTGCATATA
MtUbiquitin carboxyl-terminal hydrolase3	MtUBq Forward	GAACTTGTTGCATGGGTCTTGA
MtUbiquitin carboxyl-terminal hydrolase	MtUBq Reverse	CATTAAGTTTGACAAAGAGAAAGAGACAGA
