# Supplementary material for: MtNIP5;1, a novel Medicago truncatula boron diffusion facilitator induced under deficiency
Source: BMC Plant Biol. 2020 Dec 9;20:552. doi: 10.1186/s12870-020-02750-4 (PMC7724820; doi:10.1186/s12870-020-02750-4)
Supplement: Supplementary file 2 — Additional file 2: Fig. S1. Nitrogenase activity was determined in 4-week-old plants growing under a B gradient: control conditions (media at a final B concentration of 0,1 mM B[OH3]), B deficiency (no B supplemented into the media), or B toxic conditions (1 mM B[OH3]). Nitrogenase activity was analyzed by the acetylene reduction method and expressed as nmol of ethylene generated per hour per nodule number (left panel) or per nodule weight (right panel). Data are the Mean ± SD of two independent experiments with, at least, four pooled plants and four biological replicates (n = 4). Asterisks indicate significant differences when comparing B stress plants with B control treated plants (t-Student, “*” = p < 0.01, “**” = p < 0.001). [file 12870_2020_2750_MOESM2_ESM.pdf]

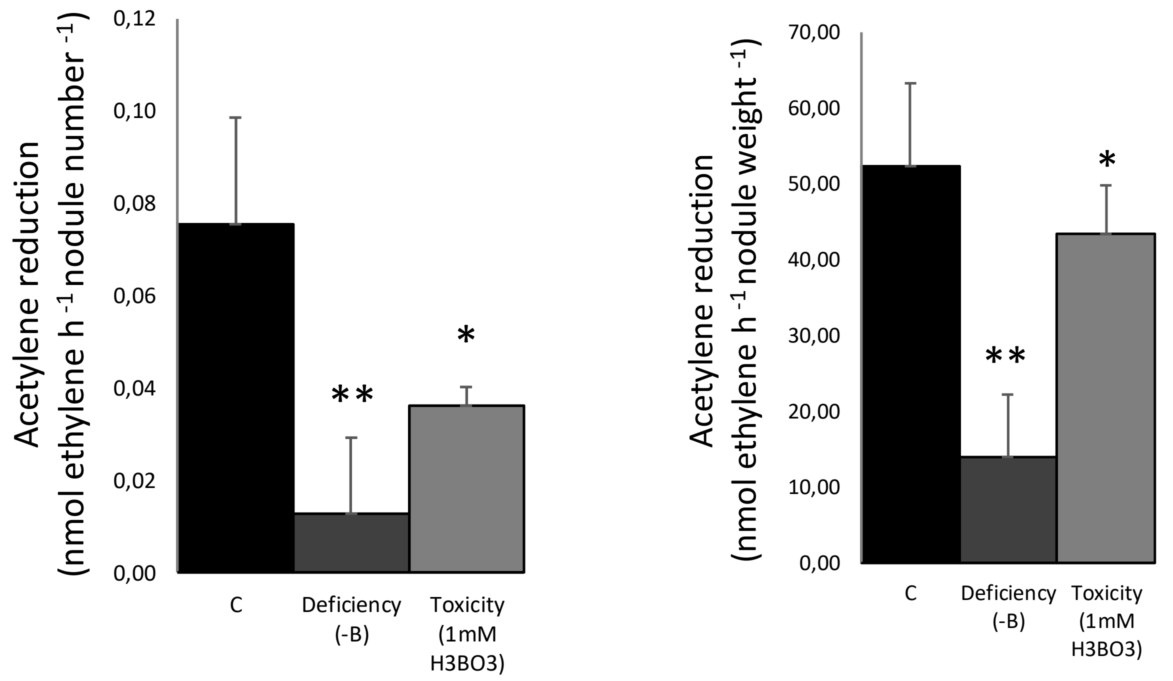

Figure S1. Nitrogenase activity was determined in 4-week-old plants growing under a B gradient: control conditions (media at a final B concentration of 0,1mM B[OH3]), B deficiency (no B supplemented into the media), or B toxic conditions (1mM B[OH3]). Nitrogenase activity was analyzed by the acetylene reduction method and expressed as nmol of ethylene generated per hour per nodule number (left panel) or per nodule weight (right panel). Data are the Mean ± SD of two independent experiments with, at least, four pooled plants and four biological replicates (n=4). Asterisks indicate significant differences when comparing B stress plants with B control treated plants (t-Student, "*"= p<0.01, "**" = p<0.001).
