# Supplementary material for: MtNIP5;1, a novel Medicago truncatula boron diffusion facilitator induced under deficiency
Source: BMC Plant Biol. 2020 Dec 9;20:552. doi: 10.1186/s12870-020-02750-4 (PMC7724820; doi:10.1186/s12870-020-02750-4)
Supplement: Supplementary file 3 — Additional file 3: Fig. S2. Expression of Medicago truncatula MtNIP5;1 (Medtr1g097840) in different plant organs. A) Data obtained from the Symbimics database (https://iant.toulouse.inra.fr/symbimics/). B) Data obtained from the Medicago Gene Expression Atlas (https://mtgea.noble.org/v3/). [file 12870_2020_2750_MOESM3_ESM.pdf]

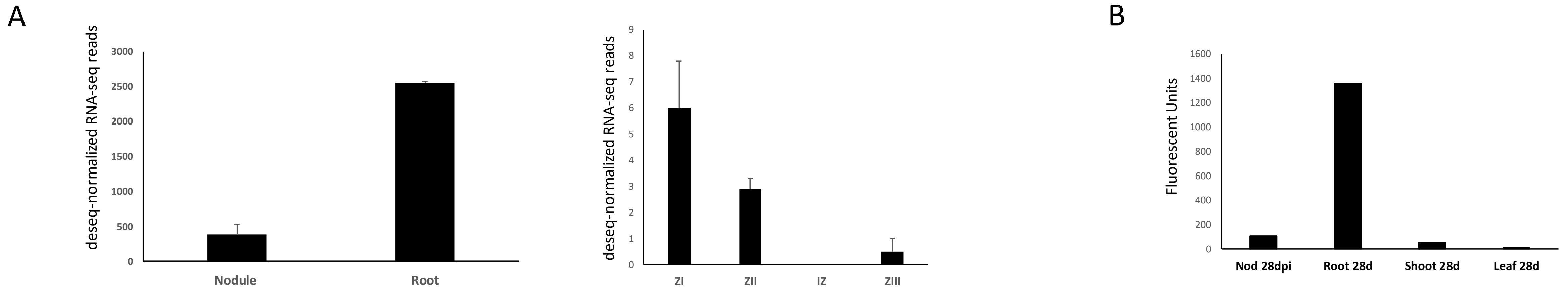

Figure S2. Expression of *Medicago truncatula MtNIP5;1* (*Medtr1g097840)* in different plant organs. **A**) Data obtained from the Symbimics database (https://iant.toulouse.inra.fr/symbimics/). **B)** Data obtained from the Medicago Gene Expression Atlas (https://mtgea.noble.org/v3/).
