# Supplementary material for: MtNIP5;1, a novel Medicago truncatula boron diffusion facilitator induced under deficiency
Source: BMC Plant Biol. 2020 Dec 9;20:552. doi: 10.1186/s12870-020-02750-4 (PMC7724820; doi:10.1186/s12870-020-02750-4)
Supplement: Supplementary file 4 — Additional file 4: Fig. S3. Time cource experiment showing primary root growth of A. thaliana seedlings in nip5; 1–1 complementation assays. Primary root growth (mm) of Wild type (Wt) (black line), two independent lines expressing p35S::MtNIP5;1-GFP construct (p35S::MtNIP5;1-GFP 1 and p35S::MtNIP5; 1-GFP 2, dark and light grey lines, respectively), and nip5; 1–1 (dashed line) were measured 3,5,7 and 10 days postgermination. Seedlings were grown under two B treatments: A) control (100 μM B[OH], and B) deficiency (0.03 μM B[OH3] [file 12870_2020_2750_MOESM4_ESM.pdf]

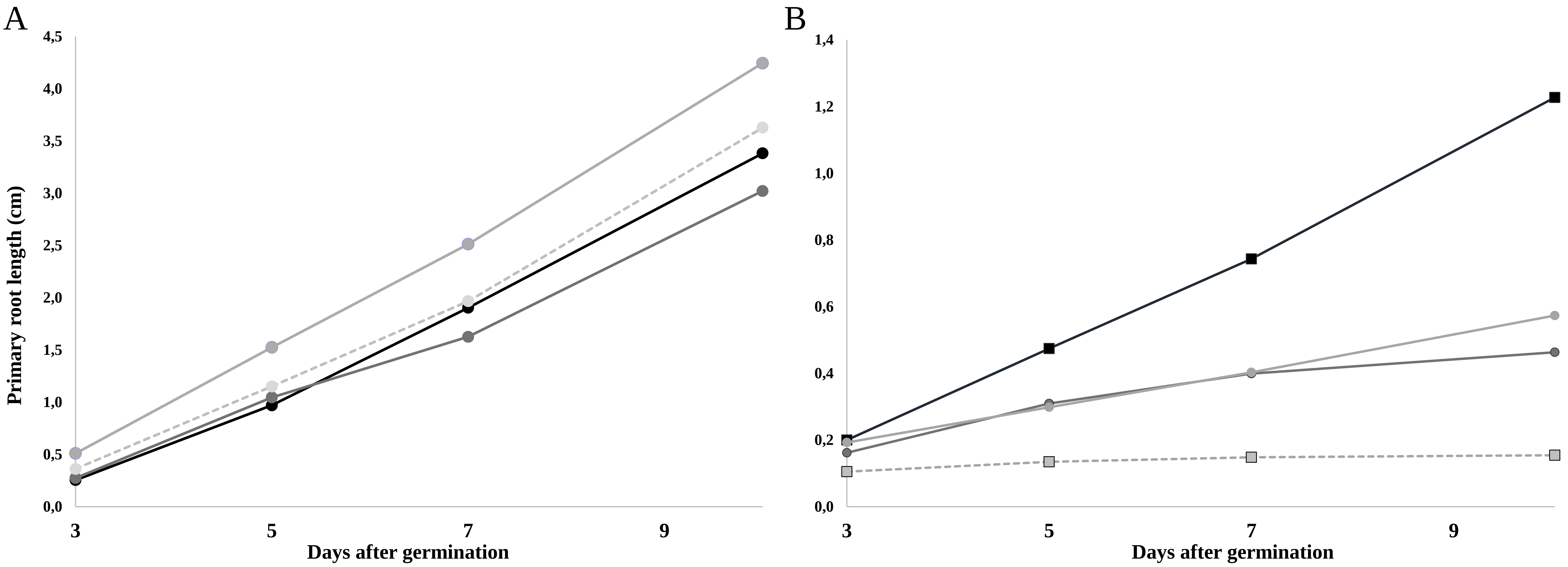

Figure S3. Time course experiment showing primary root growth of *A. thaliana* seedlings in *nip5;1-1* complementation assays. Primary root growth (mm) of Wild type (Wt) (black line), two independent lines expressing *p35S::MtNIP5;1-GFP* construct (*p35S::MtNIP5;1-GFP 1* and *p35S::MtNIP5;1-GFP 2,* dark and light grey lines, respectively), and *nip5;1-1* (dashed line) were measured 3, 5, 7 and 10 days postgermination. Seedlings were grown under two B treatments: **A**) control (100µM B[OH3], and **B**) deficiency (0.03µM B[OH3],.
