# Supplementary material for: MtNIP5;1, a novel Medicago truncatula boron diffusion facilitator induced under deficiency
Source: BMC Plant Biol. 2020 Dec 9;20:552. doi: 10.1186/s12870-020-02750-4 (PMC7724820; doi:10.1186/s12870-020-02750-4)
Supplement: Supplementary file 5 — Additional file 5: Fig. S5. Calcium (Ca) concentration was quantified in 10-days old Wild Type (Wt) (black), p35S::MtNIP5; 1-GFP 1 (dark grey), p35S::MtNIP5; 1-GFP 2 (light grey), and nip;1–1 (white) seedlings grown under B control and B deficiency conditions. The two B treatments used were: control (100 μM B[OH3] and deficiency (0.03 μM B[OH3. Different letters indicate significant differences using a One-way ANOVA analysis followed by Turkey’s test (p < 0.05). [file 12870_2020_2750_MOESM5_ESM.pdf]

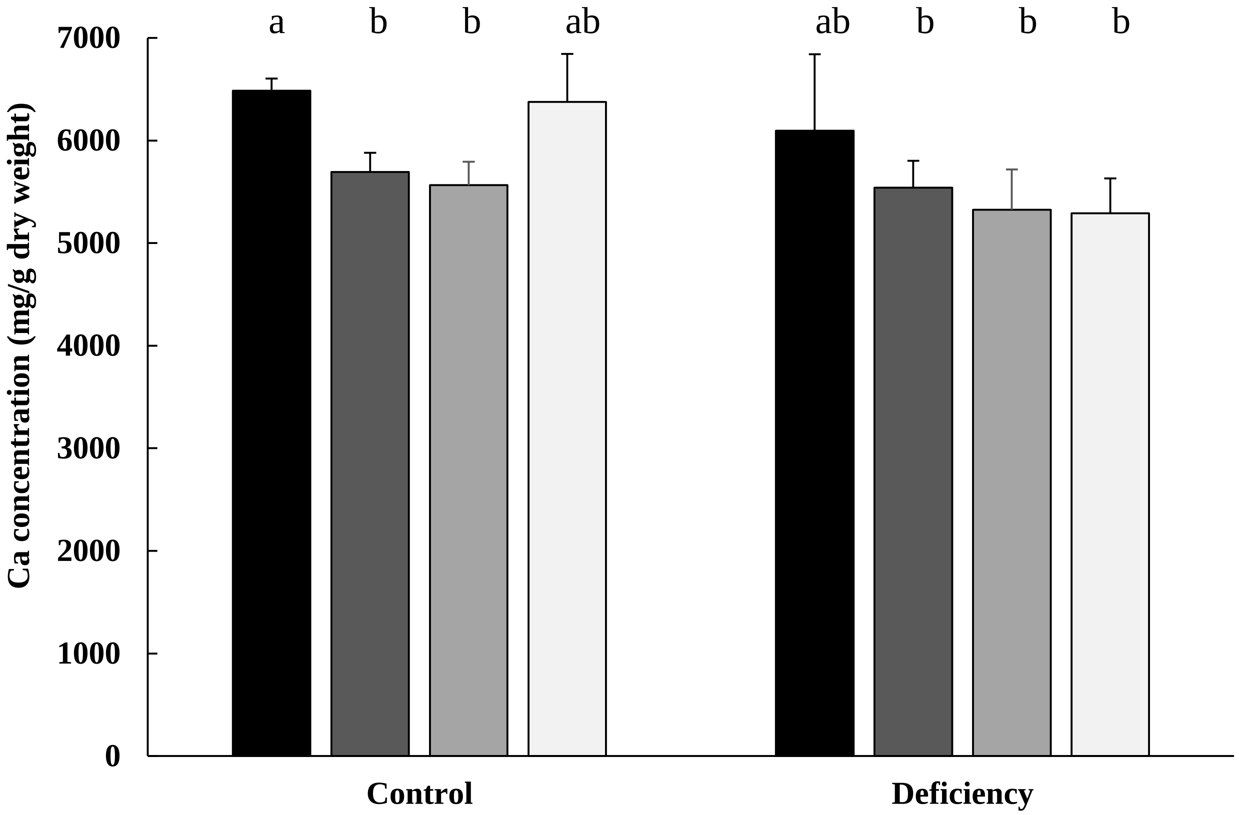

Figure S5. Calcium (Ca) concentration was quantified in 10-days old Wild Type (Wt) (black), *p35S::MtNIP5;1-GFP* 1 (dark grey), *p35S::MtNIP5;1-GFP* 2 (light grey), and *nip5;1-1* (white) seedlings grown under B control and B deficiency conditions. The two B treatments used were: control (100µM B[OH3]) and deficiency (0.03µM B[OH3]). Different letters indicate significant differences using a One-way ANOVA analysis followed by Tukey's test ($p<0.05$).
