# Supplementary material for: MtNIP5;1, a novel Medicago truncatula boron diffusion facilitator induced under deficiency
Source: BMC Plant Biol. 2020 Dec 9;20:552. doi: 10.1186/s12870-020-02750-4 (PMC7724820; doi:10.1186/s12870-020-02750-4)
Supplement: Supplementary file 6 — Additional file 6: Fig. S6.A)Sequence alignment of the UTR regions of AtNIP5;1 (AT2G47160) and MtNIP5;1 (Medtr1g097840). B) End of the 5’ untranslated region (5′UTR) of MtNIP5;1 (Medtr1g097840) and the initial sequence of the gene (starting from the ATG starting codon). [file 12870_2020_2750_MOESM6_ESM.pdf]

A

```
UTRMed      GTACTATTAAAAAATAATAATAACGCATTCATAAAACAAAAAGGAAATTTTAATTAACAA  60
UTRArabi    ------------------------------------------------------------  0

UTRMed      TATGTGCTAGCATGAGAAGAAAACGAGTAACTAGGAATATTTATAATGTATGCTCATCAT  120
UTRArabi    ------------------------------------------------------------  0

UTRMed      CAATATTAAAAAAGAAAGAAAATCATATACAAAAGAATTATTGCACAGACAATAATGCAA  180
UTRArabi    ------------------------------------------------------------  0

UTRMed      ACCCATTGACTTTTTCACATTAAGAAAATATGAAATACAAAAAGTATGGTGAATTCGTGT  240
UTRArabi    ------------------------------------------------------------  0

UTRMed      ATATTGCAAGCAAAAGCAACTTATATAGACACCATATTCATAGCACTTTCCAAACTCAAT  300
UTRArabi    -CGTTGCCAG-------------TTGCCACCAGACATACATAACACATTGAAAGCA--GC  44
               **** **             *    **   * ** **** *** **  ** *

UTRMed      TTTATAATCCATAAAACAACTTATTAATGTTAACAACTCCTCATCACTTCTTCTCCATCA  360
UTRArabi    TTATTCATCGACATACCAAACTCTCAC---------------------------------  71
            **  * *** * * * ***  * * *

UTRMed      TCCCCCTAAGTTGTTGAAAATCAACTTTTCCGACCATTAGTTCTCTCCTCCACCTTCAAC  420
UTRArabi    -----------------------------------------TCTCGCCCTCGCTCTCGCC  90
                                                     **** **  * *  **  *

UTRMed      CACGTTTTTTGCCTTTCT--------TTTTCCTATAATCTTTCCAACTTCGAAATTGGAT  472
UTRArabi    CTCGCTCTCTTTCTTTCTTCATGTAAATTGTTTGTTCTTTTCCATGTATCGAGATTCGAT  150
            * ** * * *  ******         **   * *  * ** *     **** *** ***

UTRMed      CACAACCTTCATCACAAGTTTTCTCTTATTCCATTCCAACCTTAACAACAACTACATCAT  532
UTRArabi    CTTCTTCTTCTTCACACGAACCTCCTTATTGTTTTCTCCTGCATTCTCTCTATTCTTCGC  210
            *     **** ***** *      ******   ***         *      * * **

UTRMed      CCAACTTTGATCCAATCTTTTCATATGTTCATTAATATCTTAATCCCACAATTAAATACC  592
UTRArabi    TAAACCTTGAAT------AAAGACATGTTCTTTAACATGGGTTTTTCCGGTTTAAAGTAG  264
              *** ****            * ****** **** **     *  *    *****

UTRMed      TCATCTTTCTAGCTAAATCTACAAACCTGGGTCCCTTCTTTCTTTCCATACCTTAGAAAC  652
UTRArabi    TTGTATCTTTGTTTCCCCTTTAAACCGGGTTTAAGCTCAATCTTTTCTTAGAA-------  317
            *  * * * *   *     *  ** *  *  *    **  ***** * **

UTRMed      AAATTTATAAAAACCATCCAATTATGTAACTGTACTCTTTCAACCTTAAAACTACTCGAA  712
UTRArabi    ------------CAACTCCAACTGT----------------------TTGACGATTTGAC  343
                            ***** * *                         ** * * **

UTRMed      ACAAAAAAAAAAAACAAAAAAAAATATATCTATTCTTCTTGCATTTAGAAAATTATGTAG  772
UTRArabi    TGAGAGAAAACAGAGTC-------------------------------------------  360
              * * **** * *

UTRMed      TGTTGCTAAAAATATTCACTATTTACATCCAACTATCATATTATTCTATTTTTTTTAAAG  832
UTRArabi    ------------------------------------------------------------  360

UTRMed      AAAAAAAAAAAAGA 846
UTRArabi    -------------- 360
```

B

5´UTR ← → *Medtr1g097840*

ATATTATTCTA**TTTTTTTT**AAAG**AAAAAAAAAAAAAAAAA**G**A**ATGCCAGAATCGGAGTCAGG

Figure S6. A) Sequence alignment of the UTR regions of *AtNIP5;1* (*AT2G47160*) and *MtNIP5;1* (*Medtr1g097840*). **B)** End of the 5' untranslated region (5’UTR) of *MtNIP5;1* (*Medtr1g097840*) and the initial sequence of the gene (starting from the ATG starting codon)
